# Feather characteristics of loral zone in an insectivorous passerine: The Iberian gray shrike *Lanius meridionalis*, in southern France

**DOI:** 10.1002/ece3.9482

**Published:** 2022-11-08

**Authors:** Frédéric Labouyrie

**Keywords:** beak, feather, prey, rictal bristle, shrike, south France

## Abstract

In the French Mediterranean plain, the northern extreme of its native range, the Iberian gray shrike, *Lanius meridionalis*, predominantly feeds on arthropods. Its type of loral plumage plays a key role in protecting its eyes while transporting large prey. The aims are to understand the role played by feathers in protecting the animal from various types of defensive prey. I combine an inspection of large insect prey types found on larders with a review of bird specimens found in museum collections to examine the morphometric characteristics of rictal feathers and culmen. In addition, precision photographs are used to observe the posture of the plumage in natura. I could identify four categories of protective feathers: clustered bristles, semi‐bristles, semi‐plumes distributed in the loral area, and semi‐plumes above the eyes. My results suggest that the Iberian gray shrike has a complex structure of loral feathers, specific to its foraging activity and prey types. The presence of these rictal bristles is probably a protection against the movements of larger prey items, which might damage loral zone of *Lanius meridionalis*.

## Graphical abstract

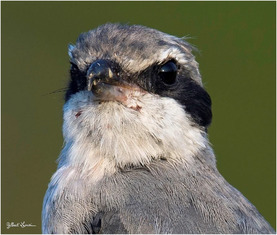

## 1 INTRODUCTION

Various studies have examined the variation in the size and shape of rictal hair‐like in birds and proposed several hypotheses as to their function (Cunningham et al., 2011; Lederer, 1972; Stettenheim, 1973). Particularly in birds who use their beaks to immobilize their prey, such as diurnal insectivorous birds of the “sit‐and‐wait” type or species that capture their prey in flight, it appears plausible that rictal hair‐like serve the function of eye protection or prey retention (Cunningham et al., 2011; Dyer, 1976).

Bristles provide eye and face protection from spiny appendages and other prey threats (Conover & Miller, 1980; Dyer, 1976; Sherry & McDade, 1982). Facial bristles are modified hair‐like feathers and are typically rigid, stiff, and tapering (Lederer, 1972).

Avian bristle feathers are found especially at the base of the bill and nostrils, as well as in the lore (between the bill and eye), malar (cheek, below the eye), and rictal (corner of the mouth) regions and forehead, and can sometime take the form of “eyelashes” (Chandler, 1914; Stettenheim, 1973).

There is a range of bristle types, from the basic structural plan of the feathers from which they are derived, through variously branched semi‐bristles, to the stiff, unbranched bristles seen around the beak commissure in many aerial insectivores (Chandler, 1914; Stettenheim, 1972, 1973). The bristle rachis is generally pointed and dark in color, particularly at the tip (Stettenheim, 1973). This dark coloration is caused by heavy deposits of melanin, which increases the strength and abrasion resistance of feather keratin (Bonser, 1996) and also contributes to bristle stiffness (Stettenheim, 1972, 1973).

The *Laniidae* spp. are a very distinctive group of small to medium size predatory passerines (14–27 cm), capable of preying on large insects and small vertebrates (lizards, rodents, and occasionally other birds). They are known for their distinctive behavior of impaling prey (up to 10 mm) on thorns and twigs for food reserves.

Their hunting technique is mostly sit‐and‐wait or perch‐and‐pounce. Usually, they approach their prey by flying at altitudes of two to three meters, sometimes briefly hovering over the prey, before quickly descending to it.

The shrike's beak is highly specialized for predaceous feeding (Cade, 1967, 1995). They execute vertebrate prey by biting the neck and disarticulating cervical vertebrates (Sustaita & Rubega, 2014). The shrikes' eyes' position is elevated and probably contributes to a wider binocular field of vision (Schön, 1996). The area between the eye and the beak is a sensitive zone when catching poisonous or urticantic food.

Types of prey vary in composition of noxious organs or secreting chemical substances, with hard and thick carapaces (Antczak et al., 2005; Labouyrie, 2022; Yosef & McPherson, 2016). The loggerhead shrike, *Lanius ludovicianus*, is able to overcome the toxic defenses of a variety of chemically defended invertebrates such as the grasshopper (*Romalea guttata*), the bella moth (*Utetheisa ornatrix*), and the beetle (*Lytta polita*) (Yosef et al., 1996; Yosef & Whitman, 1992). The same behavior has been observed in the Levantine shrike *Lanius excubitor aucheri* in Israel with the highly venomous Orthoptera *Poikylocerus bufonius*. A three‐day period likely is presumably allowed for detoxification and subsequent consumption of unsavory prey (Fuisz & Yosef, 2001). It also occurs in the Great gray shrike *Lanius excubitor* when amphibians are captured and empaled using a skinning technique (Antczak et al., 2005).

The Iberian gray shrike, *Lanius meridionalis*, is a monotypic species of the family that is geographically restricted to the Mediterranean region of the Iberian Peninsula and southern France. Given their tendency to colonize dry open environments, they are scarce at altitudes above 1000 m (Hernández, 2020).

A remarkable range of prey species are available to them, from mosquitoes and tiny ants and spiders to tetrapod vertebrates with a body mass equal to or exceeding their own. Both as an individual and as a population, the Iberian gray shrike can be very opportunistic due to its specialization in temporally and spatially limited prey abundance (Hódar, 2006).

While insects are the most common prey item of the Iberian gray shrike, their diet also includes arthropods, lizards, birds, and small mammals in Spain (Hódar, 2006).

The diet varies both regionally and seasonally. Similarly, in French habitats, insects dominate in its diet composition, with only few mammals and birds (Lepley, 1998). Regarding seasonal variations, Hymenoptera are mainly consumed in autumn, Arachnidae in autumn and winter, Orthopterae in summer and autumn, and Lepidopterae larvae in winter and spring including by young birds. Coleopterae were ingested in large numbers throughout the year. Carabidae were the main prey in winter and Melolonthidae were particularly important for adults during nestling, as were Cetoniidae for the chicks (Lepley et al., 2004).

The present study focuses on the structure of the loral plumage of the Iberian gray shrike in southern France, where they feed on arthropods which they capture primarily with their beak. Our hypothesis is that the composition of loral bristles is morphologically highly adapted for this particular type of prey capture mode, directly affecting the shape, length, and arrangement of feathers in the area between the eyes and beak.

## 2 MATERIALS AND METHODS

In order to examine the structure of rictal bristles in the Iberian gray shrike in southern France and evaluate their potential protective function against hurtful prey, we first studied larders with potentially noxious large prey species in order to assess the danger these preys may pose to the shrike's loral zone after being caught and carried in the beak.

In a second step, I examined zoologically naturalized Iberian gray shrike in the National Natural History Museum of Paris and taxidermized in life‐like poses of specimen in the Natural History Museum of Nimes (both France).

I combined them with high‐resolution photographs that show the structure and orientation of the rictal bristles in natura during arthropod capture.

### 2.1 Study of harmful arthropods found on the Iberian gray shrike larders

Since the Iberian gray shrike mainly impales its prey in winter, we focused on the period between December 2018 and the end of February 2019 to record a total of 341 prey items on a vineyard in southern France (43.810070 N, 4.201536 E; Labouyrie, 2020). Eight additional large prey were photographed in November 2021 at another location 12 km away (43.791740 N, 4.04955 E). As smaller preys (<10 mm) are eaten directly and do not appear to be impaled, I mostly found large arthropod prey items, including species with spiny parts, spines, or chemical secretions that could get into the eyes zone during handling on the floor or in larders.

### 2.2 Examination of specimens in museum collections

Bristles are a highly specialized type of feather in which the spine is relatively stiff, more tapered, and free of barbs for most of its length. Essentially, bristles are functionally simplified contour feathers that are found almost exclusively on birds' heads, and they are still clearly visible on the specimens stored in the collections.

We measured the length of the rictal bristles and beaks (distance from the tip of the upper mandible to the base of the culmen) on all available specimens from the Mediterranean plains in southern France (outside the Iberian Peninsula) registered in the collections of the Natural History Museum of Nimes and the National Natural History Museum of Paris (both France; Table 1).

**TABLE 1:** Examples of the examined specimens of Iberian gray shrikes preserved in the museums.

Natural history museum	Collection	Reference number	Origin	Date of capture
Nîmes	*Montmaison*	*358*		
	*Crespon*	*359*		
	*Crespon*	*362*		
	*Clement*	*368*		
	*Clement*	*405*		
Paris		*CG2002‐207*	France	
	*Cheylan*	*CG2022‐18*	Vauvenargues	1971
		*CG1960‐290*	St Martin de Crau	1930
	*Mayaud*	*CG1979‐132*	Bidart	1938
	*Mayaud*	*CG‐1970‐80*	Laure‐Minervois	1969
	*Debru*	*CG 1997 410*	Rongas 34	
	*N. Guillaumet*	*CG 1970–80*	Laure‐Minervois	1969
	*Potel*	*CG2000‐1776*		

**FIGURE 1:**
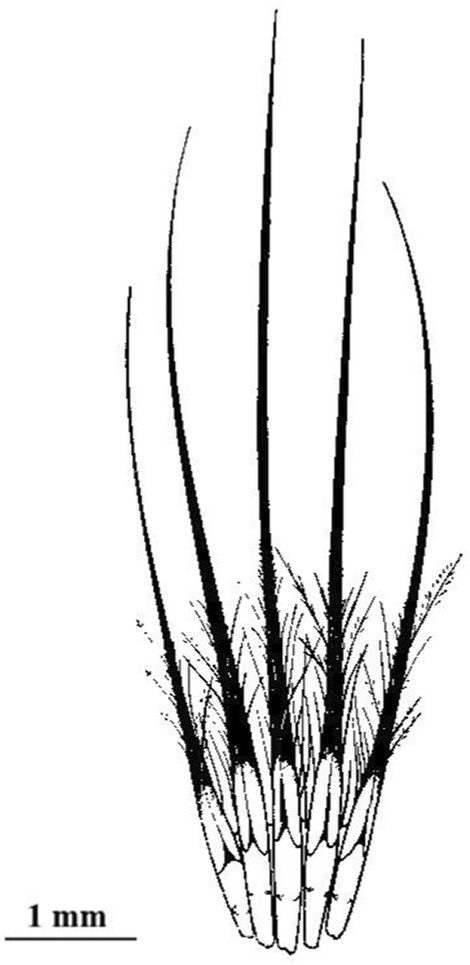
Illustration showing a cluster of rictal bristles of the Alder flycatcher *Empidinax traullii*. Seven millimeters in length; reproduced from Lederer (1972).

Rictal bristles form a cluster of four to five hairs at the base of the beak (Figure 1), and we measured the longest of these hairs with a digital caliper. The length of the beak was measured at the base of the culmen.

### 2.3 Photographic study in natura

We took high‐resolution images of the head of Iberian gray shrikes from short distances of seven to eight meters, using an ornithological blind and a progressive approach to minimize the disturbance of the birds.

## 3 RESULTS

Larders contained mostly invertebrates (Table 2). Hymenoptera accounted for 87.4% of impaled prey, of which the common bumblebee, *Bombus terrestris*, was by far the prey item accounting for 85.4% of all Hymenoptera. The second most frequent prey group were Orthoptera (7.4%), consisting of large specimens, with the migratory locust *Locusta migratoria t*he most commonly found species (3.7%). Beetles were the third most frequent prey group accounting for 3.4% of all prey collected. These are also large subjects and included *Carabus coriaceus* coprophage and Scarabaeidae such *as Bubas bubalus*. I also found some vertebrates (0.6%) such as the wood mouse *Apodemus sylvaticus* and the white‐toothed shrew *Crocidura russula*, which are particularly important for fresh biomass. Other marginal prey included the earthworm *Lumbricus terrestris* (1.2%).

**TABLE 2:** Number of prey items collected (NP) and relative frequency (RF%) in Iberian gray shrike larders in southern France between December 2018 and February 2019 and in November 2021.

Phyl./clas./ordr.	Species	NP	RF %	Dangerousness
Invertebrates				
Annelida	*Lumbricus terrestris*	4	1.1	
Arthropoda				
Hexapoda				
Orthoptera				
	*Locusta migratoria*	13		•
	*Oedipoda caerulescens*	3		
	*Anacridium aegyptium*	1		•
	*Decticus albifrons*	1		•
	*Acrididae ind*.	8		
Coleoptera				
Carabidae	*Carabus coriaceus*	2		•
Staphylinidae	*Ocypus olens*	3		•
Scarabaeidae	*Bubas bubalus*	5		•
Mantidae	*Mantis religiosa*	2		•
Hymenoptera				
	*Vespa velutina*	3		•
	*Xylocopa violacea*	4		•
	*Bombus terrestris*	298		
Vertebrates		2	0.6	
Mammalia	*Crocidura russula*	1		
	*Apodemus sylvaticus*	1		
Total		349	100	

**FIGURE 2:**
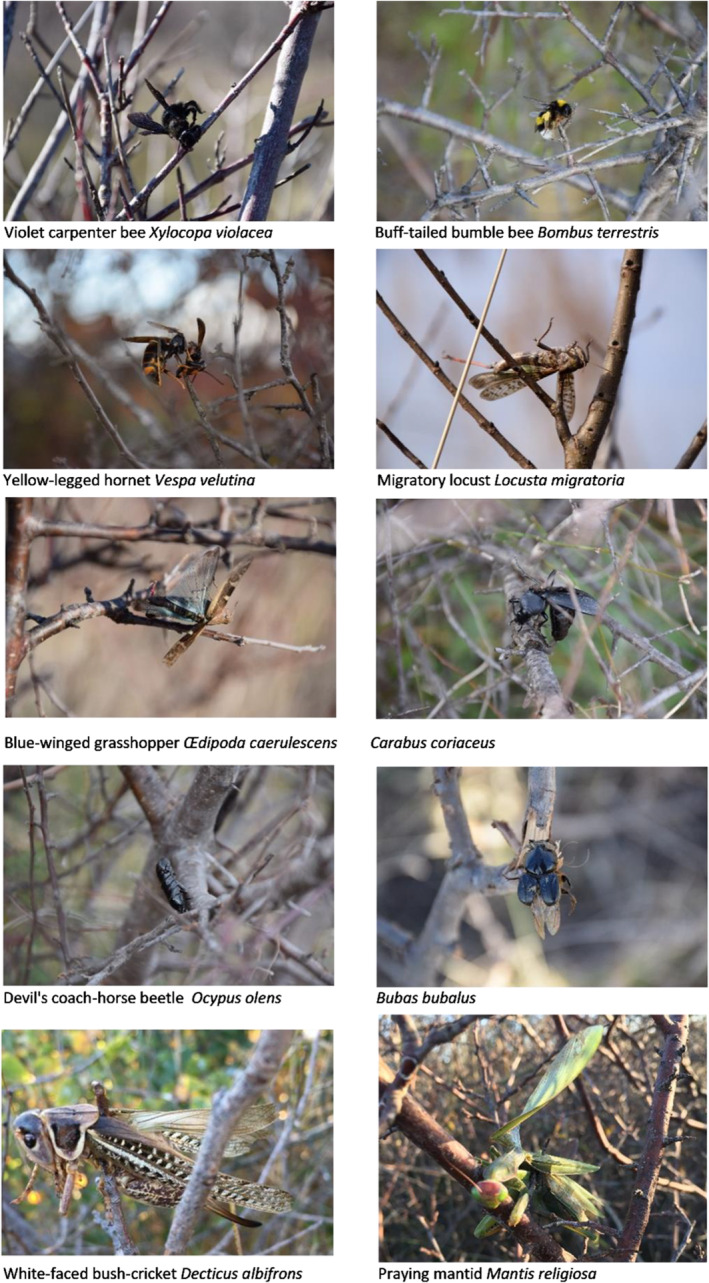
Examples of large prey found on Iberian gray shrike larders in southern France.

Among the Hymenoptera, prey observed on the larders (Figure 2) were invasive and aggressive yellow‐legged hornet, *Vespa velutina*, which was likely speared for detoxification.

There were also some large orthoptera with broad wings and jagged legs and Coleopterae with strong shells and chemical defenses. The devil's coach beetle, *Ocypus olens*, is known for raising its long and uncovered abdomen and opening its jaws like a scorpion when threatened. Though it does not have a stinger, it can deliver a painful bite with its strong, pincer‐like jaws. It also gives off a foul‐smelling odor that stems from a defensive liquid secreted by two white glands at the end of its abdomen.

I also found the praying mantid, *Mantis religiosa*, among the prey items. They possess a raptorial foreleg with unusually long coxa, which, together with the trochanter, give the impression of a femur. The femur itself is the proximal segment of the grasping portion of the leg.

**FIGURE 3:**
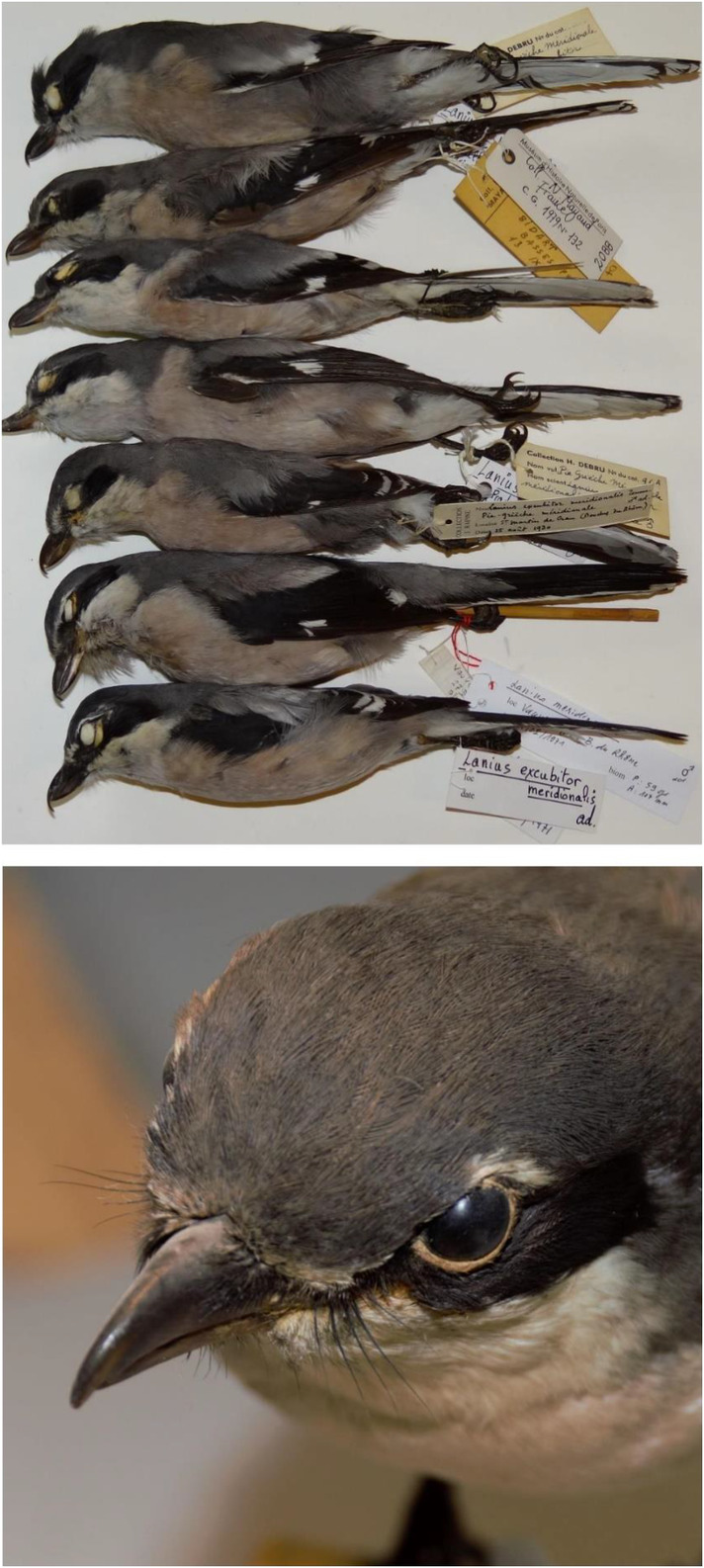
Specimens of the Iberian gray shrike (National Natural History Museum of Paris) and detail of the head of a taxidermized bird (Natural History Museum of Nîmes). © Frédéric Labouyrie.

Measurements of the bristles and beaks on museum specimens yielded the following mean lengths (±1 standard deviation; Figure 3):

Bristles: 10.27 ± 0.12 mm.
Beak: 15.96 ± 0.21 mm.
Sample size: *n* = 13.

**FIGURE 4:**
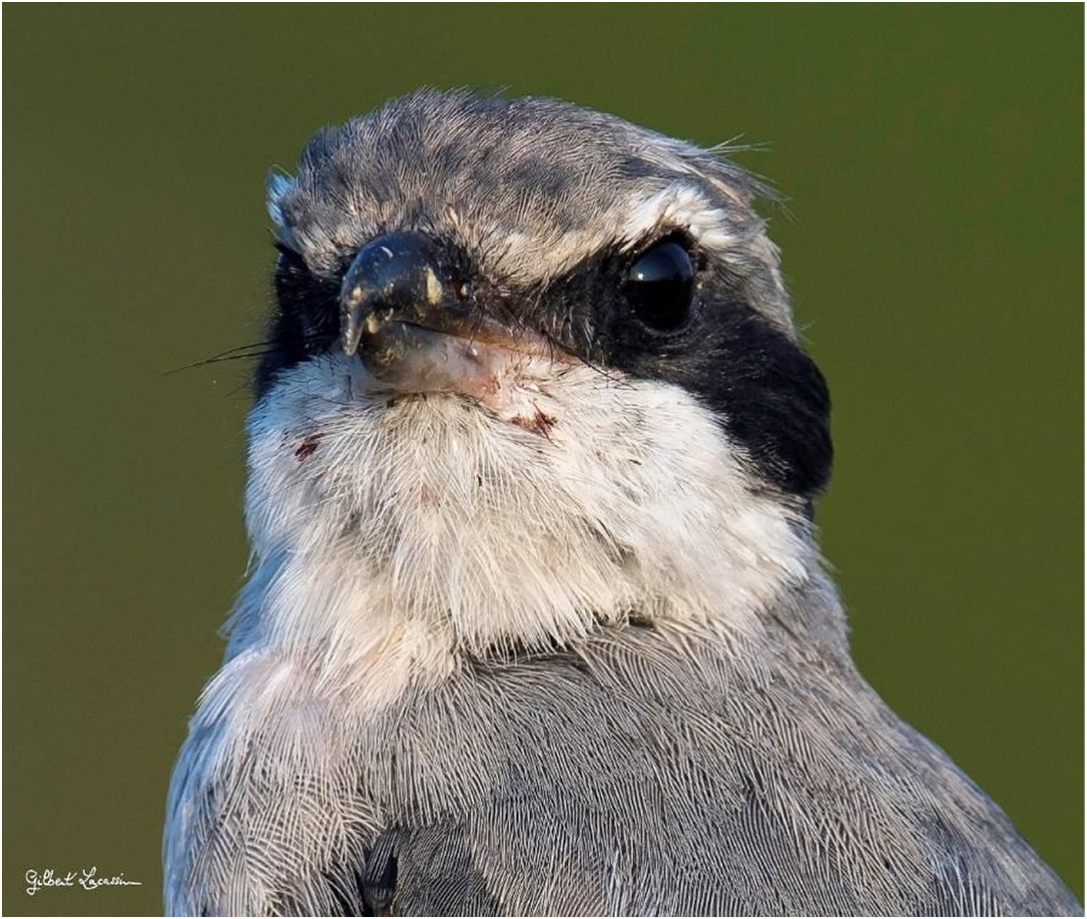
Iberian gray shrike head seen from the front. © Gilbert Lacassin.

**FIGURE 5:**
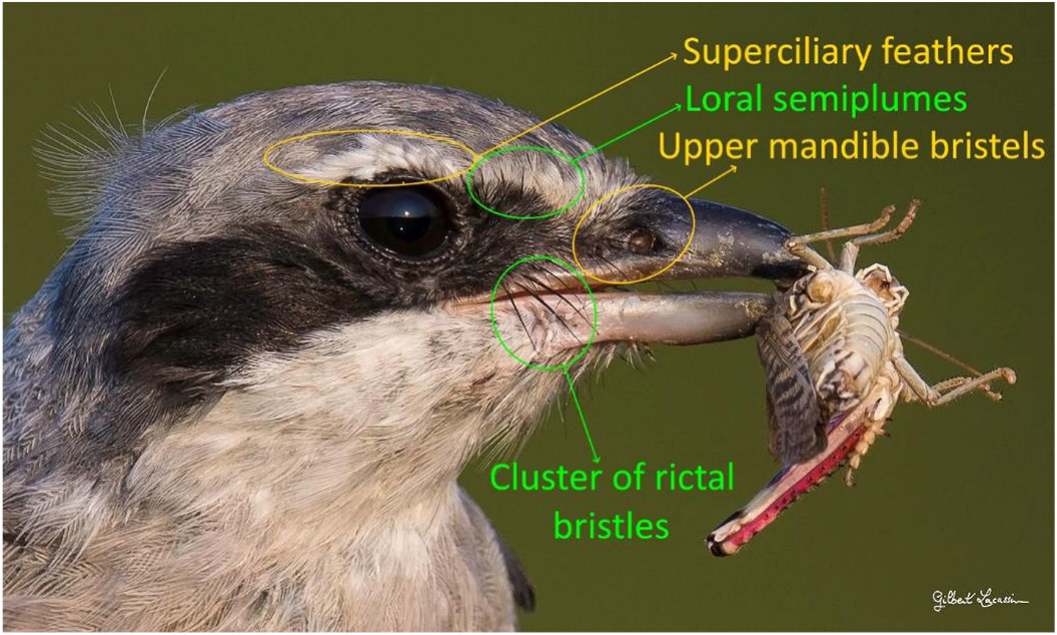
Iberian gray shrike head seen from the side showing details of the different types of protective feathers.

The high‐resolution digital photographs clearly show a row of feathers above the eyes (Figure 4). A dense network of small feathers pointing upwards, like a thick eyebrow. In the loral area, between the eye and the beak, there are feathers with black rachis and vanes ending with an open pennaceous portion with vertical barbs (Figure 5). Furthermore, there is a cluster of four strong rictal bristles with bare shafts at the commissure of the mandibles on the upper maxilla, thick, curved down, and protruding from the lower mandible (Figure 5).

In addition, a series of smaller bare shaft bristles occupy the loral region at the base of the culmen, covering a narrow band of the upper part of the mandible (Figure 5).

**FIGURE 6:**
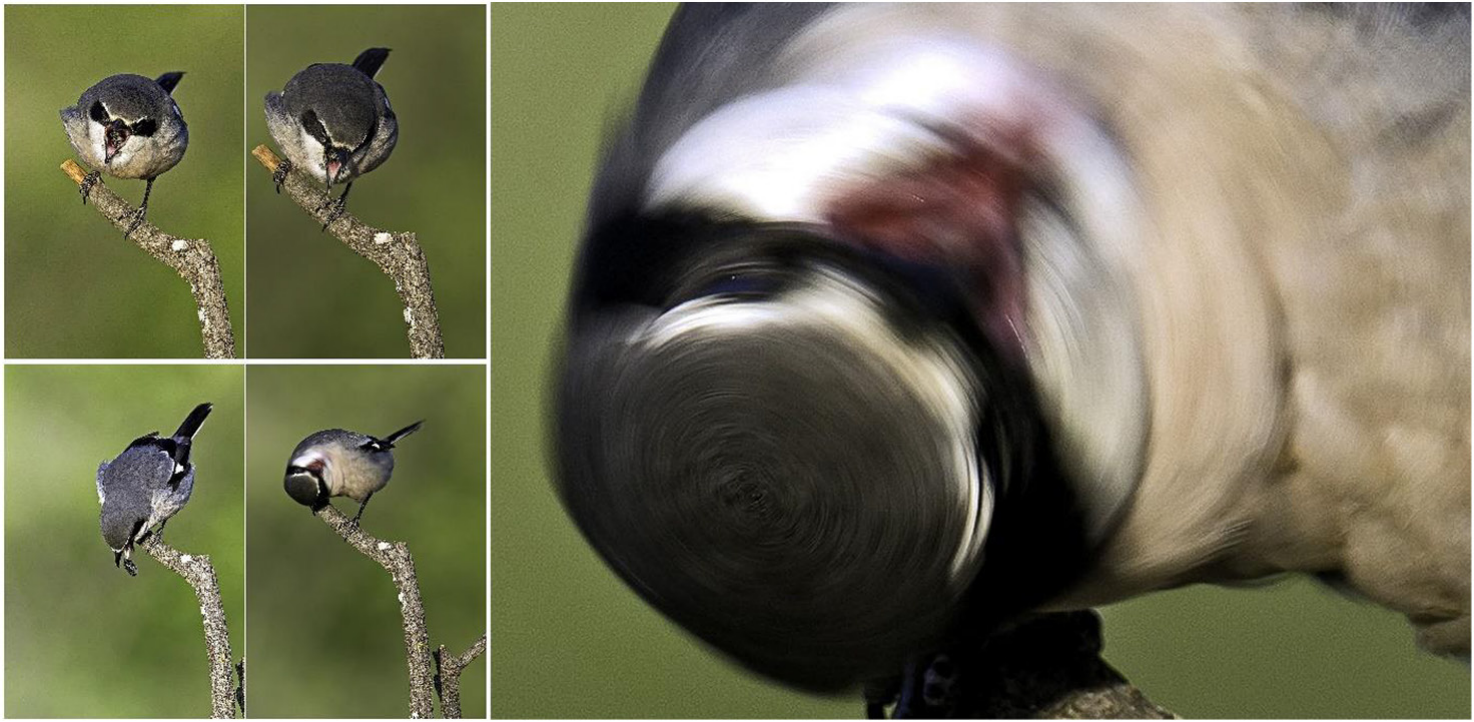
Rapid head rotation of an Iberian gray shrike to clean his face. (in this photography, after regurgitating a pellet).

During these photographic sessions, I noticed a different type of behavior related to the cleaning of the beak area. Usually, following an attack on a victim, shrikes almost always clear their bill by rubbing it against branches. In this case, during a regurgitation of a pellet, the shrike turns its to get rid of residual dirt by rotating the head very quickly (Figure 6).

## 4 DISCUSSION

The present study in no way reflects the food composition of the Iberian gray shrike in southern France. Diet composition must be determined by combined analysis of pellets and larders (Paczuska et al., 2021). But most importantly, many Orthoptera can be found in the larders.

Shrikes have often been compared to birds of prey (particularly falcons) because of their morphological and behavioral similarities. The shape of the shrike's beak bears many striking resemblances to a falcon's beak, complete with hook and tomial tooth. The slightly protruding position of the eyes facilitates binocular vision and then gives an appearance of a heavy head (Cade, 1967, 1995; Schön, 1996).

Shrikes kill prey with their beaks and carry the largest prey with them. They then hold them with their feet on a piece of vegetal support to manipulate them or impale them on a thorn bush to dismember them with their beak. In Southern France, the northern extent of its range, the diet of the southern gray shrike consists mainly of insects (Lepley, 1998; Lepley et al., 2004). Museum specimens possess a powerful beak with an average length of 15.96 ± 0.21 mm which is slightly longer than on specimens found in Spain (13.96 ± 0.64 mm, Gutiérrez‐Corchero et al., 2007).

It is well known that bill size and shape in shrike vary as a function of geography and climatic factors and may result in varying degrees of suitability for certain species of vertebrate and arthropod prey (Strong, 1901; Sustaita & Rubega, 2014). Shrikes with narrower culmen and longer hook tips produce lower bite forces than those with thicker culms and shorter hook tips (Sustaita & Rubega, 2014).

Only few bristle measurements have been carried out on the genus *Laniidae*. In long‐tailed shrike, *Lanius schach*, and great gray shrike, *Lanius excubitor*, black barbed bristles can be up to 11 mm long in the schach, and 7 mm in the excubitor. They also possess numerous shorter, barbed, black nasal bristles that curve over the sides of the beak and nostrils, before merging into loral half‐bristles (Stettenheim, 1973).

In the Iberian gray shrike, the rictal bristles are clustered into bundles of four or five strands with a maximum length of 10.27 ± 0.12 mm. They can block wings or serrated leg parts when the bird is manipulating large insects of the genera Orthopterae and Mantidae (Guillaumot, 2021) and are likely to play a role in protecting the birds' eyes when transporting large prey. Another possible function of rictal bristles that remains utterly unexplored is the detection of movements of preys caught in the beak, providing a sensory function like whiskers in some mammals (Cunningham et al., 2011).

On the loral area, there are several types of feathers: a group of rictal bristles, upper mandibular bristles, loral semiplumes and superciliary feathers. The first three types range from the basic structural level of feathers from which they are derived, to rigid, unbranched bristles, to variously branched half‐feathers. The spindle of the bristles is pointed and dark in color, especially at the base. This dark coloration is due to high levels of melanin deposition, which increases the strength and abrasion resistance of feather keratin (Bonser, 1996). It contributes to the stiffness of the bristles (Stettenheim, 1972, 1973). These three types of bristles play a protective role against injury from large prey.

In the fourth type, a row of small, tightly packed feathers forms a prominent white eyebrow above the eyes. While the shrike's skull is very similar to that of typical passerines, except for the slightly increased eye spacing, the position of the eyes in gray shrikes is somewhat peculiar, as the relatively large eyes protrude somewhat to the side of the head (Schön, 1996). This thick crown is part of the range of protective eye feathers.

Shrikes are capable of rapid axial head rolls which rotate their prey's bodies around their own necks creating accelerations equivalent to about 6 g (Sustaita et al., 2018). These accelerations are sufficient to kill mammalian vertebrate prey and by causing pathological damage to the cervical vertebrae and spinal cord. *Lanius meridionalis* also uses this technique to effectively clean out irritating particles trapped in the loral area during prey capture.

Analysis of the rictal plumage of the Iberian gray shrike shows that the species is well adapted to the capture of large prey by the beak. This allows it to have a wide spectrum of prey as well as hunting tactics.

## AUTHOR CONTRIBUTIONS

**Frédéric Labouyrie:** Conceptualization (lead); data curation (lead); formal analysis (lead); funding acquisition (lead); investigation (lead); methodology (lead); project administration (lead); resources (lead); software (lead); supervision (lead); validation (lead); visualization (lead); writing – original draft (lead); writing – review and editing (lead).

## FUNDING INFORMATION

The funding is private.

## Data Availability

Data openly available in a public repository that issues datasets with DOIs.
