# Corrigendum

**DOI:** 10.1002/ece3.9574

**Published:** 2022-12-21

In the recent article by Labouyrie (2022), the RF% values of rows “Orthoptera,” “Coleoptera,” and “Hymenoptera” are missing in Table 2.

The correct Table 2 is shown below:

**Table:** 

Phyl./clas./ordr.	Species	NP	RF %	Dangerousness
Invertebrates				
Annelida	*Lumbricus terrestris*	4	1.1	
Arthropoda				
Hexapoda				
Orthoptera	*Locusta migratoria*	13	7.4	●
	*Oedipoda caerulescens*	3		
	*Anacridium aegyptium*	1		●
	*Decticus albifrons*	1		●
	*Acrididae ind*.	8		
Coleoptera			3.4	
Carabidae	*Carabus coriaceus*	2		●
Staphylinidae	*Ocypus olens*	3		●
Scarabaeidae	*Bubas bubalus*	5		●
Mantidae	*Mantis religiosa*	2		●
Hymenoptera	*Vespa velutina*	3	87.5	●
	*Xylocopa violacea*	4		●
	*Bombus terrestris*	298		
Vertebrates		2	0.6	
Mammalia	*Crocidura russula*	1		
	*Apodemus sylvaticus*	1		
Total		349	100	

The author apologizes for the error.
